# Prediction of Material Failure Time for a Bucket Wheel Excavator Boom Using Computer Simulation

**DOI:** 10.3390/ma14247897

**Published:** 2021-12-20

**Authors:** Andrei Andraș, Sorin Mihai Radu, Ildiko Brînaș, Florin Dumitru Popescu, Daniela Ioana Budilică, Eva Biro Korozsi

**Affiliations:** Department of Mechanical, Industrial and Transport Engineering, University of Petroșani, 332009 Petrosani, Romania; sorin_mihai_radu@yahoo.com (S.M.R.); kerteszildiko@ymail.com (I.B.); fpopescu@gmail.com (F.D.P.); danalupulescu@yahoo.com (D.I.B.); koreva@gmail.com (E.B.K.)

**Keywords:** fatigue, damage, bucket wheel excavator, boom, time response, strain, stress

## Abstract

Breakdown of stackers and excavators in opencast mines is possible because of operating, manufacturing and structural causes, and it produces high financial losses. These can be prevented by using various measures, including analyses and strength tests, with computerized modeling and simulation using FEA or other techniques being implemented in the recent years. In this paper a fatigue study is conducted on the boom of a BWE. Based on a computer model of the boom previously developed in SOLIDWORKS by our author team, first the modal analysis is conducted for three positions of the boom by studying the frequency response during the excavation process. This is followed by the time response determination corresponding to the maximum displacement frequency, in order to assess the stress during the excavation process, which causes the material fatigue in the boom structure. It was found that the maximum displacements appear when the BWE boom operates in a horizontal position. The aim was to estimate the period of time to failure in order to prevent unwanted accidents, and to develop a method that is applicable to any surface mining or industrial machine with similar structure.

## 1. Introduction

Bucket wheel excavators (BWEs), along with several other heavy machines—such as spreaders and reclaimers—are used worldwide in the opencast mining industry for excavation, transportation and handling of material. This type of machines are designed, built and tested according to one of existing standards and regulations (ISO, DIN, AS, EN, etc. [1,2,3,4]) and must be able to withstand various stressful conditions of operation for decades, offering low costs of operation and at the same time high standards of safety, during their entire operational life.

In the case of BWEs, it was observed that the most vulnerable part is the boom, which is a steel structure of frames and trusses subjected to dynamic loads produced by variable excavation forces and vibrations.

There are several scholars studying the boom of bucket wheel excavators, from the point of view of estimating the remaining life, resistance to fatigue or causes that lead to its failure. Usually fatigue analysis is performed in the case of BWEs that have exceeded or are close to their usual service life.

It was found that welded joints and parts are likely to fail either because of material fatigue or defective welding process [5,6,7,8,9,10]. Other causes leading to failure in the case of BWEs are fatigue cracks of carrying structure, discharge boom ties, gusset plates, bucket wheel axle, steel slewing bearings and buckets [11,12,13,14,15,16], as well as improper design, operation conditions or manufacturing defects of parts [17,18,19].

Several methods are used to investigate the causes of failures in the case of BWEs: numerical analyses using FEM, experimental measurements, or a combination of both [20,21,22,23,24,25,26,27].

Heavy machinery and load carrying steel structures like cranes, stackers, offshore structures and steel bridges [28,29,30,31,32,33,34] are also investigated and assessed for failure and fatigue using methods similar to those used in the case of BWEs.

Based on these approaches and methods, remaining service life can be predicted and estimated [35,36,37,38] in order to propose preventive refurbishment or repairs and extension of operation if possible [39,40,41,42].

However, in comparison to the design phase of new BWEs, there are no standards when it comes to the assessment of remaining lifetime of excavators already in operation for 30 years or more, as it is the case in most open-pit mines in Eastern Europe (Poland, Serbia, Romania, etc.)

Correct determination of their technical condition, assessment of wear and fatigue of the structural material and estimation of remaining service life are of paramount importance from both economic and safety points of view, enabling further operation while ensuring proper safety and lower costs.

The study uses SOLIDWORKS Simulation, a powerful package of structural analysis tools based on FEA that provides analysis capabilities of linear, non-linear, static and dynamic nature. The step-by-step approach used in the present paper is based on the model of the ERc 1400 BWE boom, previously developed by the same authors and validated [43,44,45], and consists of the following steps:

1. The excavation forces are determined and imposed on the model in the case of a bucket wheel fitted with cutter-loader buckets;
2. Based on the estimated forces of excavation, we analyze the frequency response, considering a 2% global damping, in three positions of the BWE boom: maximum height excavation position, horizontal position and lowest possible excavation position under the bench;
3. For the horizontal position of the boom the simulations show the maximum amplitude response, so for this position we will perform the dynamic analysis of the time response with a 2% global damping;
4. As the time response results show the element with the biggest displacement for each modal frequency, we can conduct a fatigue study.

## 2. Materials and Methods

### 2.1. Description of the BWE Boom Model

**Figure 1:**
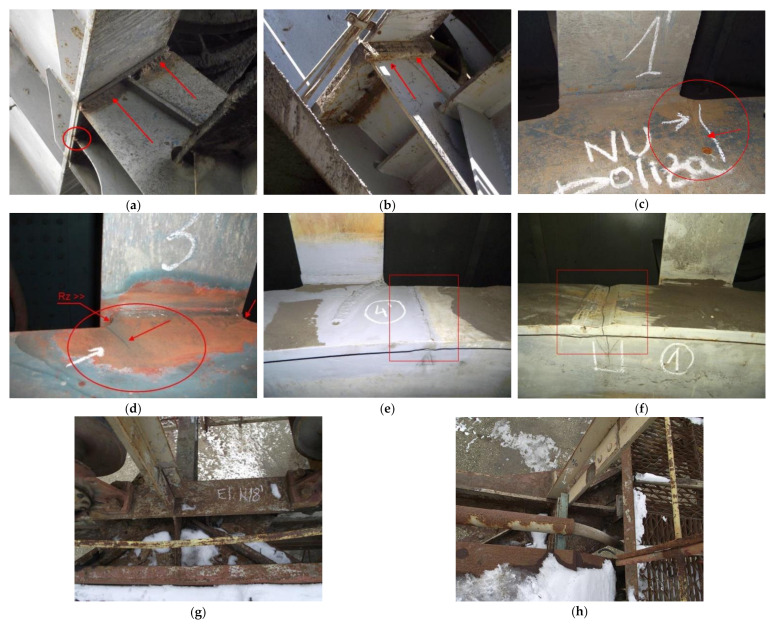
Various fissures and signs of corrosion in structural elements (**a**,**b**) Cracks of welded joints in bucket wheel support area caused by variable stresses; (**c**,**d**) Fissures caused by high roughness (Rz) value and material fatigue; (**e**,**f**) Cracks of welded joints in Heat Affected Zones (HAZ); (**g**,**h**) Corrosion of structural elements caused by exposure to environmental factors).

The analyzed BWE is the ERc-1400 30/7 model built in Romania in cooperation or under a license from Krupp. Currently, approximately 60 units of this type are working in various opencast mines belonging to the Oltenia Energy Complex—Mining Division, out of which only 28 are totally modernized. From the records of the company, most failures occurred in five of the oldest units still in operation, localized in Pinoasa and Tismana opencast mines, with 27, 29, 30, 37 and 40 years of operation. The failures are caused by fissures of structural elements due to material fatigue, welded joints failure or corrosion (Figure 1).

Taking into consideration the technical data provided by the manufacturer [46,47] for this model excavator, we developed a simplified model for the boom—bucket wheel assembly at real scale, using SOLIDWORKS (2019 Student Edition, Dassault Systèmes, Vélizy-Villacoublay, France) [43,44].

It was decided to only model these two components of the upper structure, as it is the most vulnerable subsystem, both because of the shape and constructive characteristics but also because their extremely difficult conditions of operation. These work conditions are the source of external loads with a very dynamic character, producing vibrations of cyclical variability, and possibly displacements and material fatigue and failure.

**Figure 2:**
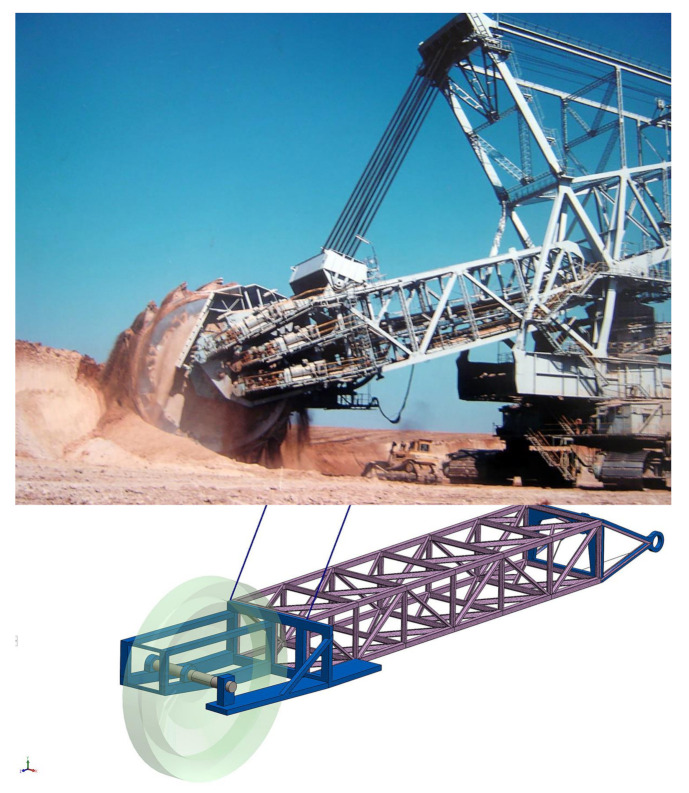
The boom of the excavator: top—real machine; bottom—developed model.

The actual boom is a three-dimensional space frame truss made of steel beams, bracings and struts connected by welded joints, which has a front section where the bucket wheel and the hoist cables are attached, a middle section with a conveyor system mounted and a back section where it is attached to the rest of the superstructure, as presented in Figure 2.

### 2.2. Excavation Forces Acting on the Bucket Wheel-Boom Assembly

**Figure 3:**
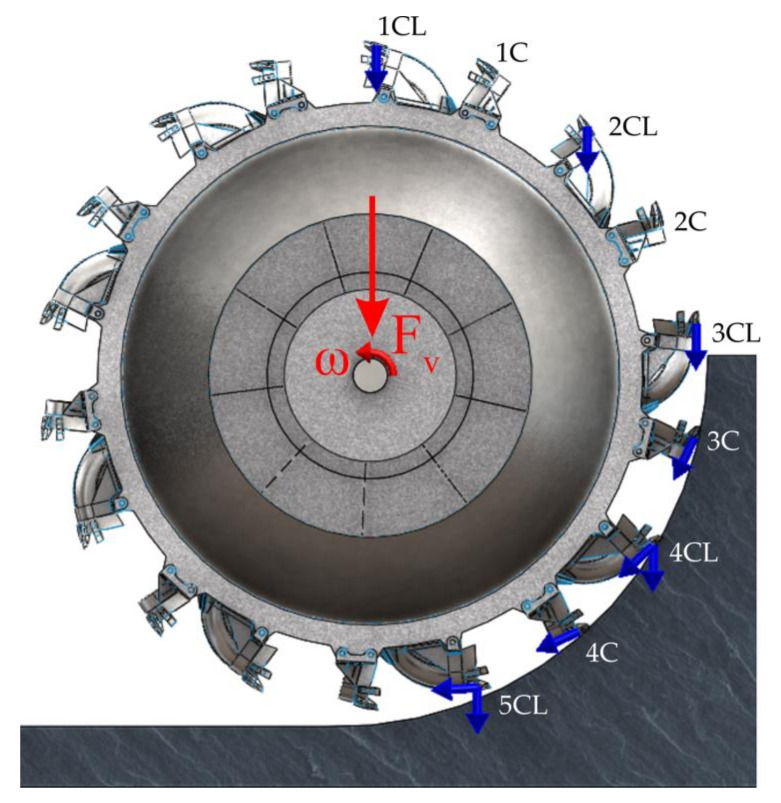
The virtual model of the bucket wheel and the forces acting on it.

The real bucket wheel has 18 buckets, half cutting and loading type and half cutting buckets only. Figure 3 presents the virtual model of the bucket wheel of the ERc-1400 30/7 excavator and the excavation forces acting on it. There are cutting and loading buckets noted with 1 CL to 5 CL and cutting buckets noted with 1 C to 4 C. Every bucket that comes into contact with the excavated face is subjected to the cutting forces that are tangent to the cutting circle. The cutting and loading buckets (1 CL to 5 CL) are also subjected to transport and inertia forces in the direction of gravitational acceleration, from the moment of contact with the face until the discharge on the conveyor. The values adopted for the cutting forces depend on the cutting resistance of the lignite and the geometry of the teeth placement on the bucket, while the transport and inertia forces depend on the density of the excavated material, the volume of the bucket, its filling ration and the loosening of the excavated material.

**Figure 4:**
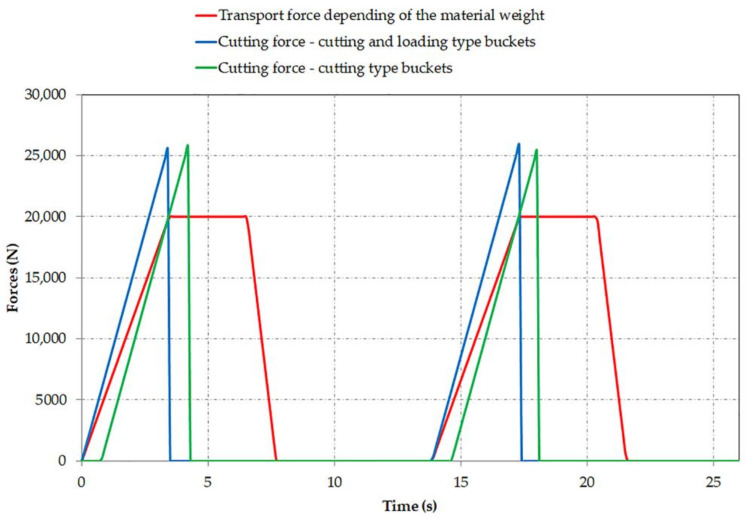
Time variation of the cutting force and transport force depending on the material.

Figure 4 shows the time variation of the cutting force for buckets 1 CL and 1 C and of the transport and inertia force for bucket 1 CL during two bucket wheel rotations. It can be observed that the first cutting bucket comes into contact with the face with a delay of 0.77 s compared to the first cutting and loading bucket. This delay is dependent on the bucket wheel characteristics (number, type and placement of buckets) and rotation speed.

**Figure 5:**
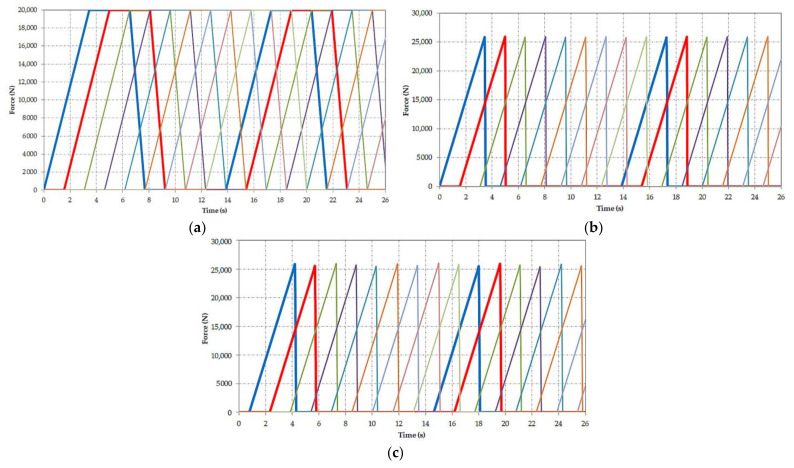
Time variation of (**a**) the transport and inertia force for all cutting and loading (CL) buckets; (**b**) the cutting force for all cutting and loading (CL) buckets; (**c**) the cutting force for all cutting (C) buckets.

Figure 5a–c show the variation diagrams of the forces for all buckets for the entire simulation period.

The simulation of the excavation process was performed by operating the bucket wheel model, using a SOLIDWORKS Motion studies motor with a constant imposed rotational speed of 4.33 rpm, which is the actual bucket wheel speed, corresponding to a frequency of around 39 buckets/minute. The total simulation time was 27.71 s, which ensures two complete rotations. We emphasize that in the simulation process this virtual motor has the role of ensuring a constant rotation for the bucket wheel, the torque and the power from its shaft being calculated using SOLIDWORKS Motion Study (Motor torque, Power consumption tools).

According to [48] (pp. 82–83), the lateral component of the cutting force has a magnitude that can be determined if the wheel speed and the slewing speed are known, but it can be ignored. Since in this case it has an even smaller value as compared to Raspers’ example, it was considered that the lateral component does not influence the results of dynamic analyses and fatigue study.

The authors of [49] moved the tangential and normal components of the resistance-to-excavation load to the center of gravity of the bucket wheel and drive unit and used an in-house-developed application to calculate and plot the main forces and moment vectors.

**Figure 6:**
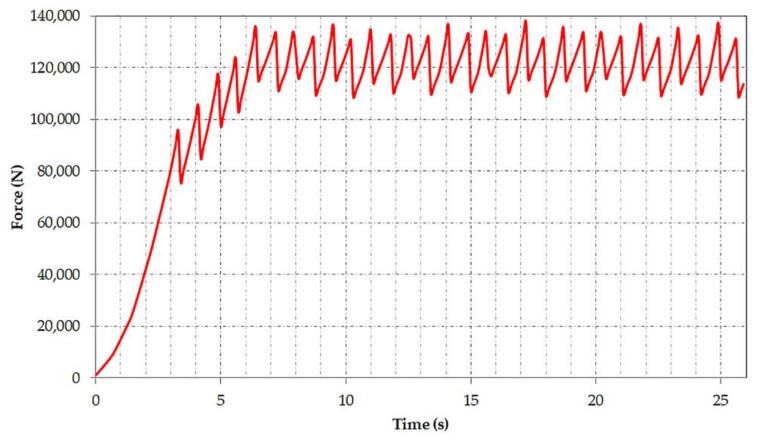
The variation in time of the vertical force.

Using this hypothesis and imposing the values and mode of variation in time of the forces as presented in Figure 4 and Figure 5a–c, we determined the variation in time of the vertical force acting on the shaft of the bucket wheel as can be seen in Figure 6.

**Figure 7:**
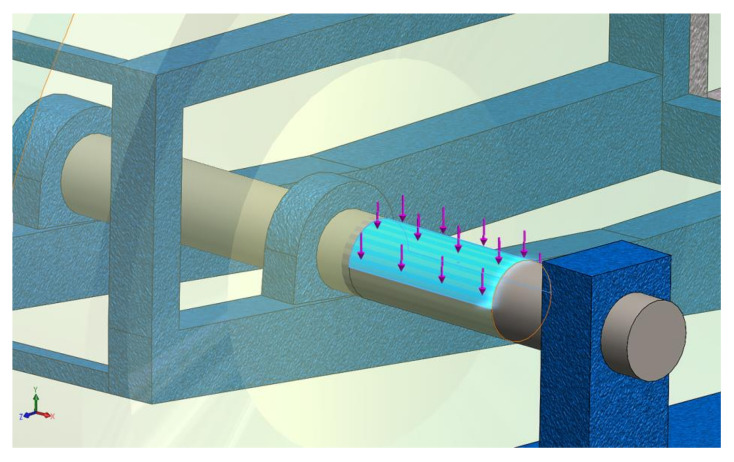
Imposing the total resultant force to the model.

The variation of the vertical force acting on the bucket wheel shaft will generate the periodic dynamic load that will form the basis of the BWE boom fatigue study. At the start, there is a transient regime up to approx. 6.4 s, until the permanent excavation regime of the BWE is reached. This force is imposed [50,51] to the model as shown in Figure 7, and it is necessary in order to conduct the dynamic response analysis in both frequency and time domain.

**Figure 8:**
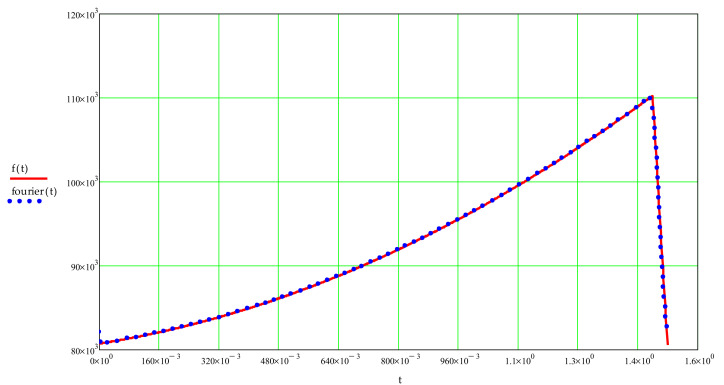
Force variation using Fourier series development for one period.

**Figure 9:**
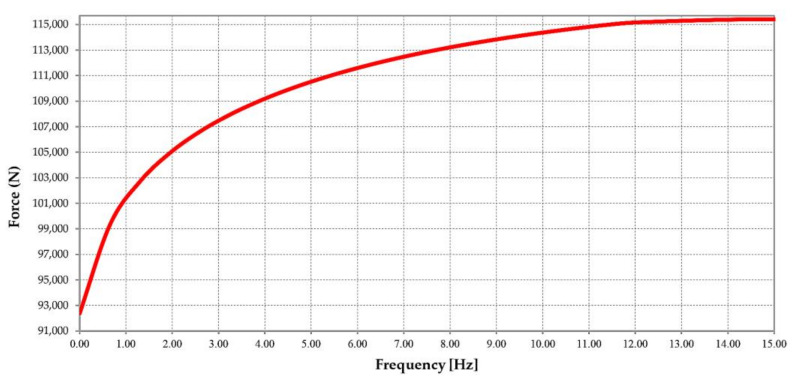
Variation of force vs. frequency.

This force is expressed in relation to frequency, so the force variation for only one period was approximated using Fourier series development (Figure 8) for frequencies up to 15 Hz as no significant force variation is observed after this value. The forces’ variation in relation to the frequency is shown in Figure 9.

**Figure 10:**
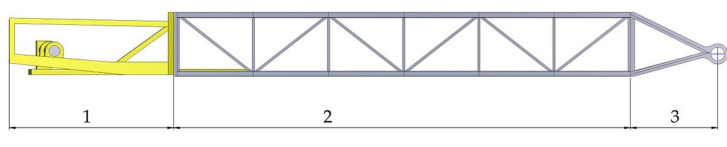
Sections of the bucket wheel boom model.

Similar to the real boom, the virtual model of the BWE boom is a three-dimensional space frame truss made of Beams, Solid and Shell type elements. It is also divided into three sections [43], as shown in Figure 10:

- Section 1 is the bucket wheel support where also the hoist cable connections and the bucket wheel drive systems are mounted;
- Section 2 is the middle section with the discharge conveyor system;
- Section 3 is the joint section of the bucket wheel boom to the rest of the superstructure.

In the model of the BWE boom, the middle section is made up a top and a bottom chord and a left and a right girder being build using Beam elements in SOLIDWORKS. Section 1 and 3 are built using Solid and Shell elements. For the beam type finite elements, the connectivity made at the nodes between the sides of the wireframe geometry is essential.

The nodes of the beam network are treated as joints for clearance equal to zero. In addition to the Global Contact bonded automatically by SOLIDWORKS based on the connections between the parts of the assembly, contacts were set between the surfaces of the beam structure but also between the nodes (joints) of the beam structure and the surfaces of the Solid and Shell type elements with which they come into contact. Without these contacts, the boom assembly subjected to dynamic or static simulations becomes unstable.

### 2.3. Static Loads Acting on the Bucket Wheel-Boom Assembly

In addition to the dynamic forces exerted on the bucket wheel, there are several static loads acting on the BWE boom itself, which are all taken into consideration during the modeling and simulation. Table 1 summarizes these static loads, their magnitude and the SOLIDWORKS feature used to model them.

**Figure 11:**
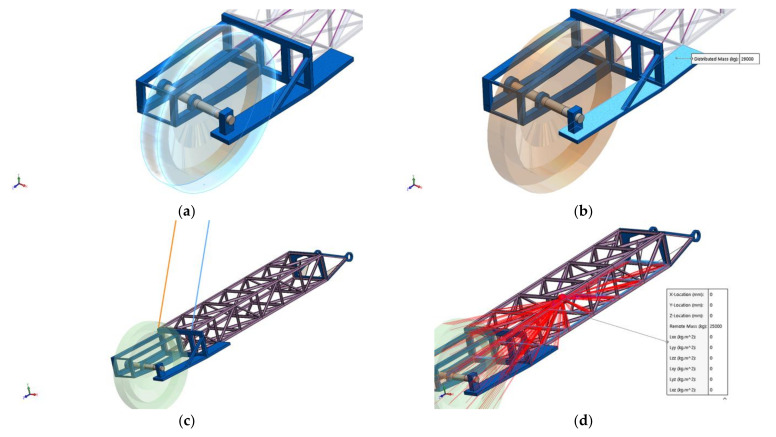
Static loads acting on the boom. Model and placement: bucket wheel weight (**a**); bucket wheel drive system weight (**b**); hoisting cables (**c**) and discharge conveyor belt (**d**).

The static load induced on the boom by the actual weight of the bucket wheel was modeled using a SOLIDWORKS assembly with real size and weight, placed on the boom front part (Figure 11a). The drive system of the bucket wheel (electric motor, gear, shaft, couplings, etc.) was modeled as a distributed mass, placed on the side of the boom as shown in Figure 11b. The actual boom is supported and moved vertically by 10 hoisting cables (Class 6 × 36 WS Steel Core Wire Rope type) which are modeled using 2 SOLIDWORKS springs with equivalent spring constant of 35 × 10^6^ N/m each as shown in Figure 11c. Finally, the discharge conveyor belt inside the BWE boom was modeled as a remote mass placed as displayed in Figure 11d.

### 2.4. Analyses Performed on the Model: Frequency Response Analysis, Time Response Analysis and Fatigue Study

As stated in the research topic steps defined in the introduction, once the model is created and forces defined, the next step is the frequency response analysis, considering a 2% global damping.

Frequency response analysis is used to obtain the response of a structure to a forced excitation, and it is used in general for low excitation frequencies with slow variation. This will be simulated and the results presented for three excavation positions of the BWE boom that can be achieved during operation: maximum height position, horizontal position and lowest possible position (under the bench).

Next, for the position of the boom where the frequency response analysis shows the maximum amplitude of the displacement, the dynamic analysis of the time response is simulated, also considering a 2% global damping.

The results of time response analysis are used as strains imposed on the BWE boom in order to determine the stress variation and values. Based on these, finally a fatigue study is conducted for a period of up to 30 years to find the moment when the damage rate reaches 100%.

## 3. Theoretical Aspects of Frequency Response

Noting with ${£}_{2d}$ the second order linear differential operator

(1) $${£}_{2d}=a_{0}\frac{d^{2}}{dt^{2}}+a_{1}\frac{d}{dt}+a_{2}$$

where *a*_0_, *a*_1_ and *a*_2_ are positive constants, the equation of the frequency response has the form:

(2) $${£}_{2d}\left\{y\left(t\right)\right\}\;=a_{0}\frac{d^{2}y}{dt^{2}}+a_{1}\frac{dy}{dt}+a_{2}y\left(t\right)\;=x\left(t\right)$$

with the formal solution:

(3) $$y\left(t\right)\;={£}_{2d}^{-1}\left\{x\left(t\right)\right\}$$

The solution *y(t)* of Equation (2) contains two integration constants independent of the excitation *x(t)*, which are determined from the conditions regarding the initial values of displacement and velocity. The solution *y(t)* of Equation (2) is called the *complete response* and is equal to the sum of two terms:

(4) $$y\left(t\right)\;=y_{sl}\left(t\right)+y_{e}\left(t\right)$$

where the term $y_{sl}\left(t\right)$ is the general solution of the homogeneous equation:

(5) $$a_{0}\frac{d^{2}y_{sl}}{dt^{2}}+a_{1}\frac{dy_{sl}}{dt}+a_{2}y_{sl}\left(t\right)\;=0$$

with the initial conditions:

(6) $$y_{sl}{\left(t\right)}_{\left|t=0\right.}=y_{sl}\left(0\right)\;\mathrm{and}\;\frac{dy_{sl}}{dt_{\left|t=0\right.}}=y_{sl}^{\prime }\left(0\right)$$

and is called the *free response to zero excitation*.

The term $y_{e}\left(t\right)$ is the general solution of the nonhomogeneous equation:

(7) $$\frac{d^{2}y_{e}}{dt^{2}}+a_{1}\frac{dy_{e}}{dt}+a_{2}y_{e}\left(t\right)\;=x\left(t\right)$$

with the initial conditions:

(8) $$y_{e}\left(0\right)\;=0\;\mathrm{and}\;y_{e}^{\prime }\left(0\right)\;=0$$

and it is called *response to zero excitation and initial state*.

In order to determine the free response to zero excitation, Equation (5) will be expressed as:

(9) $$\frac{d^{2}y_{sl}}{dt^{2}}+2\alpha \frac{dy_{sl}}{dt}+\omega_{0}^{2}y_{e}\left(t\right)\;=0$$

where α is the *damping coefficient* and $\omega_{0}$ the *resonant pulsation* or resonant cyclic frequency:

(10) $$\alpha =\frac{a_{1}}{2\cdot a_{0}}\;\mathrm{and}\;\omega_{0}=\sqrt{\frac{a_{2}}{a_{0}}}$$

The roots of the characteristic polynomial $p\left(\lambda \right)$:

(11) $$p\left(\lambda \right)\;=\lambda^{2}+2\cdot \alpha \cdot \lambda +\omega_{0}^{2}$$

are called *natural pulsations* or natural cyclic frequencies:

(12) $$\lambda_{1,2}=-\alpha \pm \sqrt{\alpha^{2}-\omega_{0}^{2}}$$

Depending on the values of the parameters α and $\omega_{0}$, there are four regimes of variation in time of the free response, namely:

**The aperiodic regime** in which $\alpha >\omega_{0}$ and the natural pulsations $\lambda_{1}$ and $\lambda_{2}$ have real negative values, and thus $y_{sl}\left(t\right)$ can be written as:

(13) $$y_{sl}\left(t\right)\;=\left(C_{1}e^{\lambda_{1}t}+C_{2}e^{\lambda_{2}t}\right)h\left(t\right);\;\lambda_{1}<0;\;\lambda_{2}<0$$

Constants *C*_1_ and *C*_2_ are determined from initial conditions Equation (6) as:

(14) $$C_{1}=\frac{1}{\lambda_{1}-\lambda_{2}}\left(y_{sl}^{\prime }\left(0\right)-\lambda_{2}y_{sl}\left(0\right)\right)\;\mathrm{and}\;C_{2}=\frac{1}{\lambda_{2}-\lambda_{1}}\left(y_{sl}^{\prime }\left(0\right)-\lambda_{1}y_{sl}\left(0\right)\right)$$

Thus $y_{sl}\left(t\right)$ is:

(15) $$y_{sl}\left(t\right)\;=\left[\frac{y_{sl}^{\prime }\left(0\right)}{\lambda_{1}-\lambda_{2}}\left(e^{\lambda_{1}t}-e^{\lambda_{2}t}\right)+\frac{y_{sl}^{}\left(0\right)}{\lambda_{1}-\lambda_{2}}\left(\lambda_{1}e^{\lambda_{1}t}-\lambda_{2}e^{\lambda_{2}t}\right)\right]h\left(t\right)$$

**The critical aperiodic regime** in which $\alpha =\omega_{0}$ and the natural pulsations have equal and negative values $\lambda_{1}=\lambda_{2}=-\alpha$. The response $y_{sl}\left(t\right)$ can be written as:

(16) $$y_{sl}\left(t\right)\;=\left(C+C^{\prime }t\right)e^{-\alpha t}h\left(t\right)$$

Constants *C*_1_ and *C*^’^ are determined from initial conditions Equation (6) as:

(17) $$C=y_{sl}\left(0\right);\;\mathrm{and}\;C^{\prime }=y_{sl}^{\prime }\left(0\right)+\alpha y_{sl}\left(0\right)$$

Thus $y_{sl}\left(t\right)$ becomes:

(18) $$y_{sl}\left(t\right)\;=\left[y_{sl}\left(0\right)+\left(y_{sl}^{\prime }\left(0\right)+\alpha y_{sl}\left(0\right)\right)t\right]e^{-\alpha t}h\left(0\right)$$

**The damped oscillation regime** in which $\alpha <\omega_{0}$ and the natural pulsations have complex values $\lambda_{1}=\;-\alpha +j\omega_{d}\;\mathrm{and}\;\lambda_{2}=\;-\alpha -j\omega_{d}$, and thus $y_{sl}\left(t\right)$ can be written as:

(19) $$y_{sl}\left(t\right)\;=Ke^{-\alpha t}\sin\left(\omega_{d}t+\beta \right)h\left(t\right)$$

where $\omega_{d}=\sqrt{\omega_{0}^{2}-\alpha^{2}}$ is called the pulsation or cyclic frequency of its own oscillation and constants K and β expressed as:

(20) $$K=\sqrt{y_{sl}^{2}\left(0\right)+\frac{1}{\omega_{d}^{2}}{\left[y_{sl}^{\prime }\left(0\right)+\alpha y_{sl}\left(0\right)\right]}^{2}};\;\beta =\arctan\frac{y_{sl}\left(0\right)\omega_{d}}{y_{sl}^{\prime }\left(0\right)+\alpha y_{sl}\left(0\right)}$$

The oscillating regime in which α=0 and the natural pulsations have imaginary values $\lambda_{1}=j\omega_{0}\;\mathrm{and}\;\lambda_{2}=j\omega_{0}$, and thus $y_{sl}\left(t\right)$ can be written as:

(21) $$y_{sl}\left(t\right)\;=K_{0}\sin\left(\omega_{0}t+\beta_{0}\right)$$

with constants *K*_0_ and $\beta_{0}$ expressed as:

(22) $$K_{0}=\sqrt{y_{sl}^{\prime }\left(0\right)+\frac{y_{sl}^{\prime 2}\left(0\right)}{\omega_{0}^{2}}}\;\mathrm{and}\;\beta_{0}=\arctan\frac{y_{sl}\left(0\right)\omega_{0}}{y_{sl}^{\prime }\left(0\right)}$$

Except for the oscillating regime the system is stable for t→∞. The oscillating regime determines a boundary stability of the system. The stability of the system responses presupposes that the real part of the complex frequency is negative, Re(λ)≤0.

The oscillating regime is characterized by the parameter called *quality factor* which is expressed as:

(23) $$Q=\frac{\omega_{0}}{2\alpha }$$

To the four regimes described above, the following quality factor values are corresponding: $Q<\frac{1}{2}$ aperiodic regime, $Q=\frac{1}{2}$ critical aperiodic regime, $Q>\frac{1}{2}$ damped oscillating regime and Q→∞ oscillating regime.

## 4. Computer Simulations, Results Obtained and Discussion

### 4.1. Frequency Response Analysis of the BWE Boom Model

In the case of dynamic frequency response analysis, it is assumed that the load applied to the structure is an explicit function of frequency, mass and damping properties. Its general equation can be expressed as:

(24) $$\left[M\right]\ddot{d}+\left[C\right]\dot{d}+\left[K\right]d=F\left(A\cos\left(\omega t\right)+B\sin\left(\omega t\right)\right)$$

where [*M*] is the mass matrix, [*C*] the damping matrix, [*K*] the stiffness matrix, *F(t)* is the vector of nodal loads expressed as a function of frequency and *d* is an unknown vector of the nodal displacements [52,53].

Since this type of analysis (frequency response) is based on mode superposition principle, it is necessary to define a global damping [54,55] expressed as a percentage of critical damping. For the analysis presented in this paper, we imposed a constant global damping of 2% which is specific to this type of structure [56].

**Figure 12:**
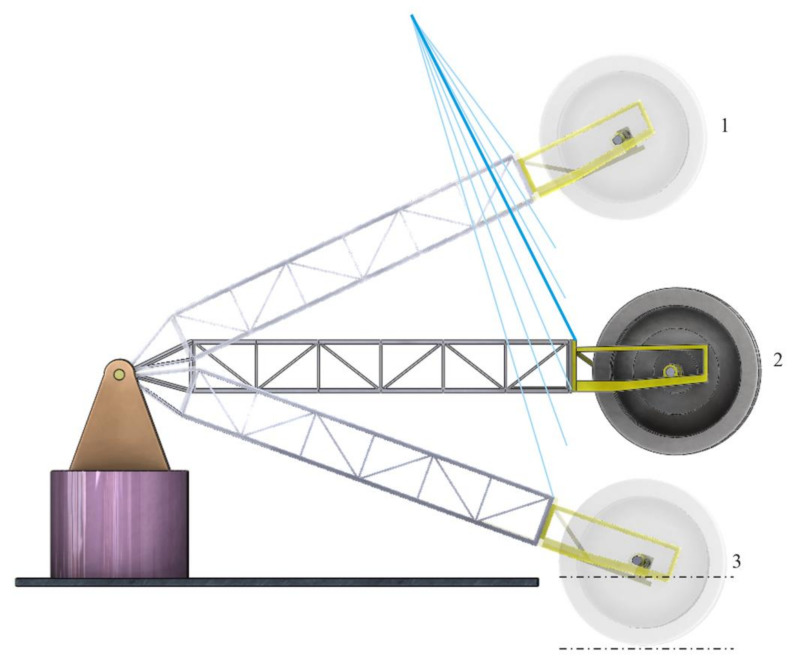
Positions of the BWE boom for which the frequency response analysis is performed: (1) the maximum height excavation position (25°), (2) the horizontal excavation position (0°), and (3) the lowest possible excavation position, under the bench (−20°).

Using the BWE boom model presented in Section 2.1 and considering the force of excitation variation as obtained in Section 2.2 the frequency response analysis was performed, for three different positions of the BWE boom:

(1) the maximum height excavation position (25°);
(2) the horizontal excavation position (0°), and
(3) the lowest possible excavation position, under the bench (−20°);

These positions can be seen in Figure 12.

The results obtained for the amplitude of the displacement are presented comparatively for every position of the boom and directions *X*, *Y* and *Z*. For all further references, direction *X* corresponds to the transverse, direction *Y* to the vertical plane and direction *Z* to the longitudinal plane.

**Figure 13:**
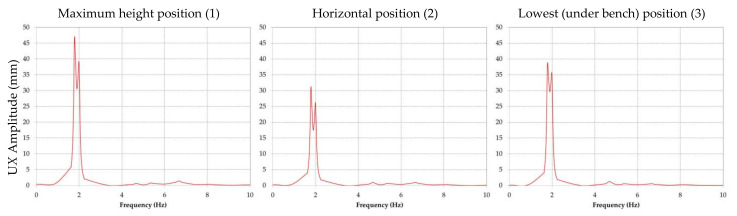
Comparative results of the amplitude of the displacement in direction *X* for positions (1), (2) and (3) of the BWE boom.

Figure 13 shows the amplitudes of the displacement and corresponding accelerations in direction *X* for the three positions considered.

**Figure 14:**
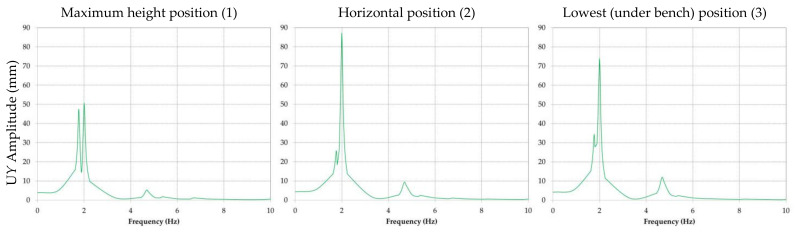
Comparative results of the amplitude of the displacement in direction *Y* for positions (1), (2), and (3) of the BWE boom.

**Figure 15:**
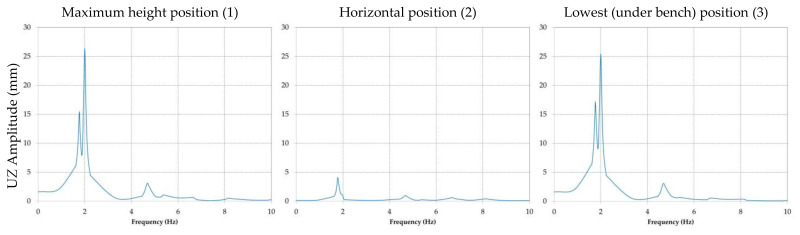
Comparative results of the amplitude of the displacement in direction *Z* for positions (1), (2) and (3) of the BWE boom.

In Figure 14 the amplitudes of the displacement in direction *Y* are shown for each of the three analyzed position, while Figure 15 presents the amplitude of the displacement in direction *Z* for the same three positions.

It is visible that the maximum amplitude of the displacement will appear in the case of the horizontal position of the BWE boom; it is in *Y* direction and was calculated by the software as being approximately 88 mm.

**Figure 16:**
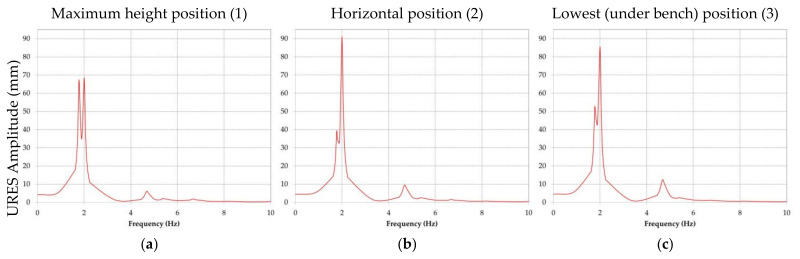
Comparative results of the resultant amplitude of the displacement for all directions, positions (1), (2) and (3) of the BWE boom.

Taking into consideration the resultant force (all three directions *X*, *Y* and *Z*), Figure 16 presents the amplitude of the resultant displacements for positions: maximum height excavation (Figure 16a), horizontal excavation position (Figure 16b) and the lowest (under the bench) excavation position (Figure 16c).

The results obtained from the simulation are summarized in Table 2. The table contains the values of the maximum displacements and their corresponding frequency, for the 15 modes considered in the dynamic frequency response analysis.

For each mode there are three groups of values highlighted, which correspond to the maximum height excavation position of the boom (25°), the horizontal excavation position of the boom (0°) and the lowest possible excavation position of the boom (−20°). The SOLIDWORKS software allows the localization of the point where the maximum displacement occurs and the visualization of this displacement at displacement scales automatically set by the software.

**Figure 17:**
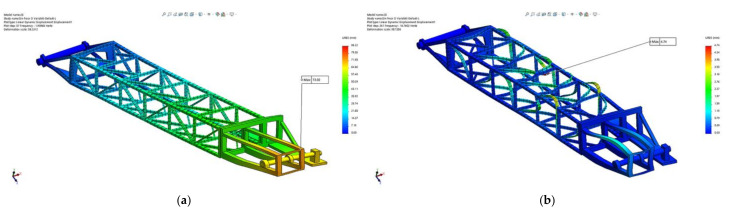
Location and shape of displacement of the boom for the maximum height excavation position (1). (**a**) Mode 2, frequency of 1.91 Hz; (**b**) Mode 12, frequency of 16.76 Hz.

**Figure 18:**
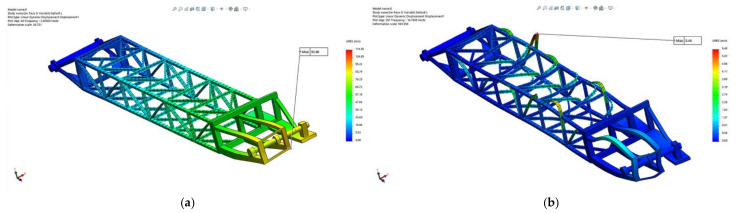
Location and shape of displacement of the boom for the horizontal excavation position (2). (**a**) Mode 2, frequency of 2.00 Hz; (**b**) Mode 12, frequency of 16.76 Hz.

**Figure 19:**
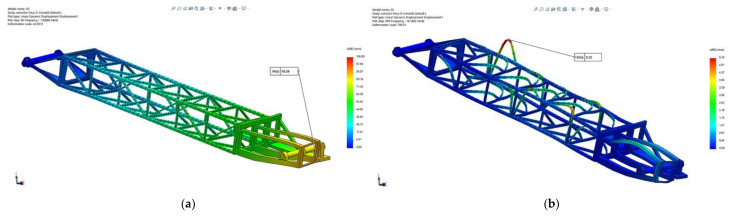
Location and shape of displacement of the boom for the lowest possible excavation position, under the bench (3). (**a**) Mode 2, frequency of 1.91 Hz; (**b**) Mode 12, frequency of 16.76 Hz.

The results show that, for all three positions of the boom taken into consideration, the maximum displacement occurs in section 1 (bucket wheel support) of the BWE boom for modes 1 to 5, and in section 2 (middle section) for modes 6 to 15. The absolute values are shown in Table 2, with section 1 having a maximum displacement of 92.6 mm, corresponding to mode 2 (1.91 Hz), when the boom is in horizontal position, while section 2 has a maximum displacement of 5.48 mm, corresponding to mode 12 (16.76 Hz), also when the boom is in horizontal position. The location and shape of these displacements are shown in Figure 17 for the maximum height position of the boom, Figure 18 for the horizontal position of the boom and Figure 19 for the lowest position of the boom.

### 4.2. Time Response Analysis of the BWE Boom Model

Time response analysis assumes that the load applied to the structure is an explicit function of time, mass and damping properties with the characteristic equation:

(25) $$\left[M\right]\ddot{d}+\left[C\right]\dot{d}+\left[K\right]d=F(t)$$

where [*M*] is the mass matrix, [*C*] the damping matrix, [*K*] the stiffness matrix, *F*(*t*) is the vector of nodal loads expressed as a function of time and *d* is an unknown vector of the nodal displacements [52,53].

The results will show the response for both the period the load is exerted as well as the free vibration, after the load is removed.

For the horizontal position of the BWE boom, where the maximum amplitude of displacement was determined to appear in *Y* direction, the dynamic time response analysis was conducted on the boom, under the same assumptions and with a 2% damping. The results of this are accelerations and displacements in all three directions [43,44] and their corresponding frequency.

In order to validate the results obtained from the time response analysis, experimental data were also recorded. In [57] a comparative analysis of vibration spectra of the same kind of boom was performed, based on acceleration measurements using MicroStrain G-Link LXRS accelerometers and MSR145 data loggers placed on the boom of an ERc 1400 excavator from Husnicioara mine and stress measurements using strain gauges.

**Figure 20:**
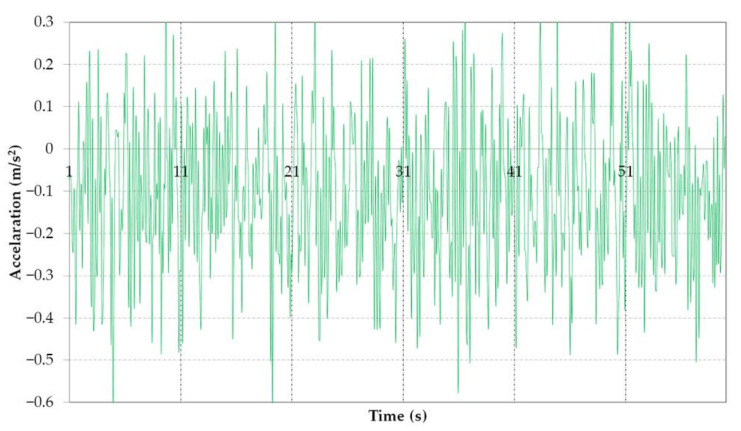
Sample of measured acceleration in vertical plane (direction *Y*).

The results obtained validate the idea that in the first section of the boom, near the bucket wheel, the low frequencies produce large displacements in the vertical direction, due to the deflection of the entire boom, while in the second section of the boom towards the third section, near the connection with the mast, the higher frequencies are dominant, and the amplitudes are smaller, represented as local displacements of the boom elements. A sample of the accelerations in vertical plane (*Y*), measured during excavation with the boom in horizontal position, saved as .csv processed and analyzed is shown in Figure 20.

**Figure 21:**
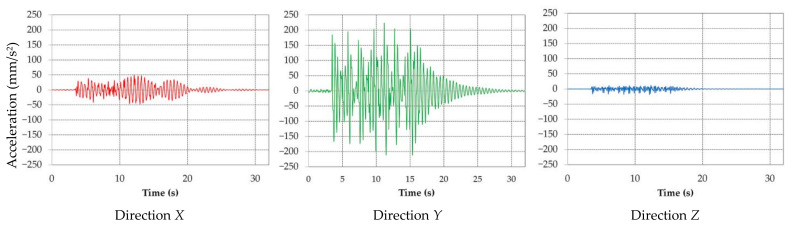
Acceleration for direction *X*, *Y* and *Z* resulting from the time response analysis.

**Figure 22:**
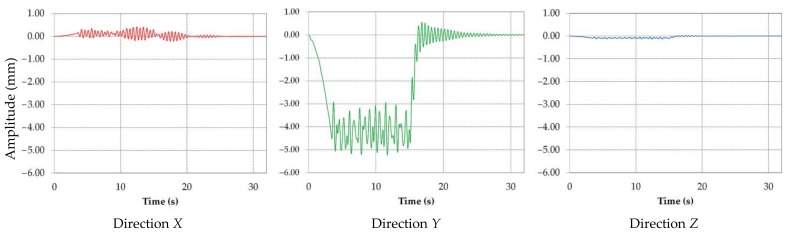
Displacement for direction *X*, *Y* and *Z* resulting from the time response analysis.

It was determined that the biggest measured accelerations also appear in the vertical plane (similar to the simulation) and it is visible that the values are close to the ones resulted after the time response analysis. The accelerations and displacement for all direction, as resulted from simulation, are shown in Figure 21 and Figure 22. The accelerations obtained after the analysis are also close to accelerations measured experimentally in [58,59,60].

**Figure 23:**
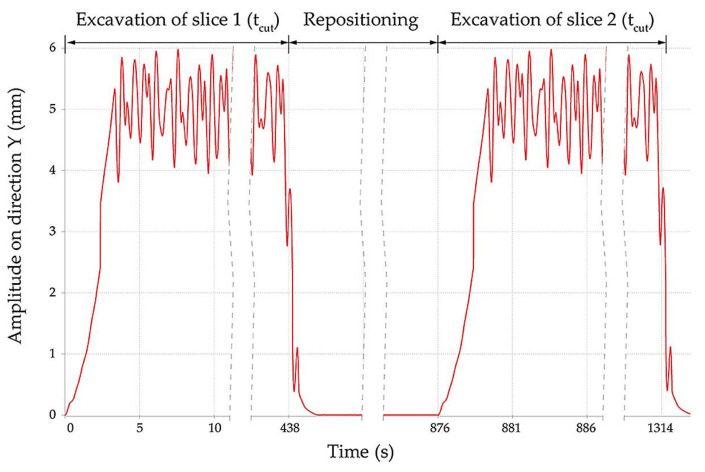
Displacement of the boom in vertical plane vs. time for two cuts.

The dominant displacement appears in the vertical plane with the resultant average amplitude of 5.2 mm and a corresponding frequency of approximately 2 Hz. The shape of the amplitude vs. time is illustrated in Figure 23 for two cuts.

This figure also shows the slewing time for the excavation of the first slice t_cut_ = 438 s, followed by repositioning of the BWE and then the time needed for a second slice, with the total time based on the geometric characteristics of excavation (slewing angles, radius etc.) for this BWE model as determined in [61]. This average displacements resulting from the time response analysis are in accordance with experimental strain gauge measurements [36,57,62]; these are used as values for the input data, as strains acting on the boom, with the boom treated as a solid in a SOLIDWORKS static simulation. The fatigue analysis requires the stress determination.

### 4.3. Fatigue Analysis

**Figure 24:**
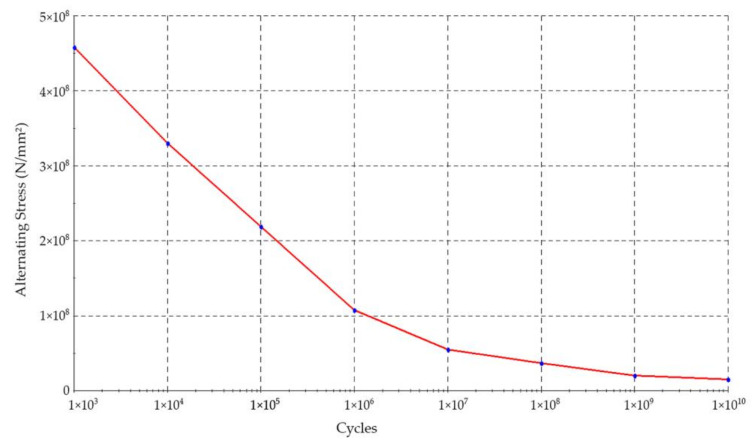
S–N curve defined in SOLIDWORKS. This experimental validation is presented in detail in [63] where the results of strain gauge measurements are compared with FEM analysis performed on a BWE working in similar conditions.

In order to perform the fatigue analysis of the truss structure of the excavator’s boom using SOLDWORKS, an S–N curve must be provided as input. In this respect, we adopted the fatigue curve in Figure 24, as determined in [62], cited in [42].

The S–N curve has been established by the regression method of the Basquin’s equation, for two representative points, related to the ultimate strength of the material- S_u_, considering for 1000 cycles S_m_ = 0.9 · S_u_ and the endurance limit S_e_′ = 0.5 · Su, considered for 10^6^ cycles. The values taken in consideration were S_u_ = 510 MPa, S_m_ = 459 MPa and consequently, S_e_′ = 255 MPa. This last one (S_e_′) has been corrected for a number of recommended factors such as size, load, surface finish, temperature and reliability, thus obtaining for the global correction factor of 0.345 the value of 87.9755 MPa for the corrected endurance limit, S_e_. Based on these, the authors determined by regression the parameters of the S–N curve, respectively, S(N) = 2195.42 · N^−0.22657^.

Since the studied BWE is of the same type, located in the same open pit mine and operating under similar circumstances, this curve was adopted to be introduced in SOLIDWORKS.

The used S–N curve has been validated for a member of the boom structure with known material characteristics, the operating time/number of loading cycles resulted from the operating time of 24.4 years (3.75 × 10^9^ cycles) and the value of stress measured on site through strain gauges during the excavation process of 17.1 MPa.

Fatigue Analysis in SOLIDWORKS (Simulation/Fatigue) has as mandatory precondition the existence of a static study as input data, besides the known parameters like number of cycles, asymmetry coefficients of the stress cycle, etc. The output of Fatigue Analysis are the estimated percentage of failure for the input cycles (Damage), the total estimated Life expectancy which predicts the life above or below the specified number of cycles and the highlight of areas of emergence and propagation of cracks leading to failure. Various parameters are also important when setting up a Fatigue Analysis, like choosing the correct S–N Curve for the material, taking the fatigue strength reduction factor K_f_ into consideration, the type of stress and the mean stress correction used, which in our case was the Gerber correction, recommended for ductile materials like steel.

**Figure 25:**
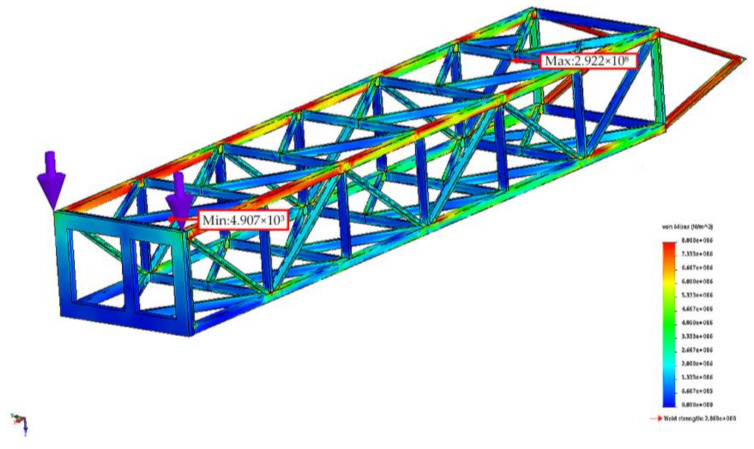
von Misses stress of the BWE boom based on the static analysis.

The fatigue study was conducted on the middle section of the BWE boom beam structure because here are the most common occurrences of Damage. The calculation of the static load was performed with the beam network of the boom treated as Solid in SOLIDWORK. The two stresses imposed at the tip of the boom are of the Reference Geometry type. They are referenced to the upper plane of the boom and have a value of 5.2 mm as resulted from the time response analysis in Section 4.2. In static load analysis, in order to highlight only the contribution of the excavation process on beam structure, we did not consider the gravitational acceleration as External Load. The stress variation and values as resulted from the static analysis are shown in Figure 25. It can be seen that the maximum obtained value of 2.922 × 10^8^ N/m^2^ is greater than the material yield stress value of 2.068 × 10^8^ N/m^2^. The highest percentage of damage is expected to appear in this point where the calculated stress exceeds the yield stress.

The total number of cycles producing damage for one year of operation can be calculated as:

(26) $$N=f_{d}\cdot t_{y}\cdot C_{iu}$$

where *f_d_* is the frequency corresponding to the maximum displacement, *t_y_* is the total operation time of the excavator in one year and *C_iu_* is the coefficient of intensive use with a value of 0.5 for this type of BWE. For normal operation $t_{y}=8\cdot 22\cdot 12\cdot 3600=7.60\times 10^{6}$ seconds, thus

(27) $$N=2\cdot 7.60\times 10^{6}\cdot 0.5\approx 7.6\;\mathrm{million}\;\mathrm{events}/\mathrm{year}$$

In SOLIDWORKS we specified the events producing the fatigue, which is the static load generated by the two reference geometries. The number of cycles was imposed based on the period analyzed and the load type Equivalent Stress (von Misses). The fatigue analysis of the BWE boom was performed for the period from 10 to 20 years a fatigue study was run at every 5 years and from 20 to 30 years a fatigue study was run every year. The exact results are presented in Table 3 in form of min/max Damage rate, period analyzed in years and events according to (27).

**Figure 26:**
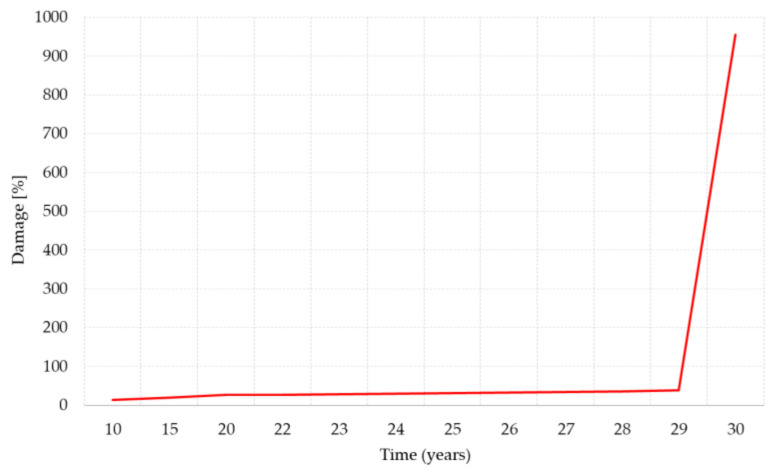
Actual variation of damage (%) max.

The initial damage rate shows a slow linear increase from 13 to 38% and then presents a significant jump after 29 years reaching the maximum Damage rate of 955.30% at 30 years. The actual variation of the max damage in percentage is plotted in Figure 26.

**Figure 27:**
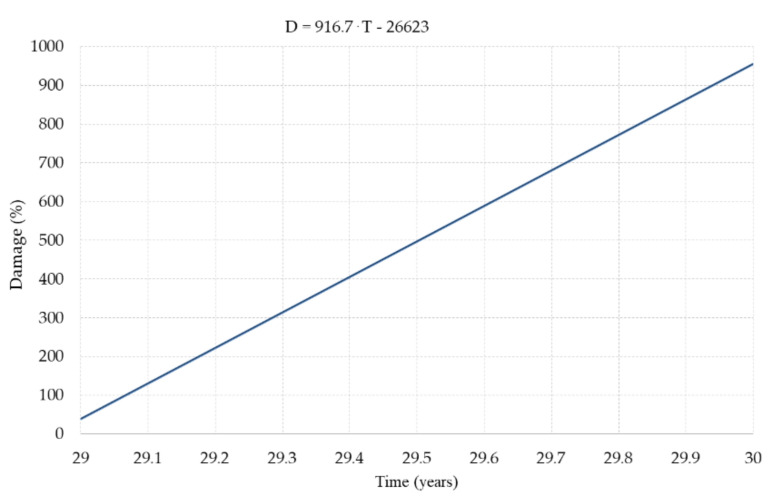
Damage variation line for 29 to 30 years period.

To capture the moment when the damage reaches 100% the damage variation line for 29 to 30 years is plotted in Figure 27 and expressed as:

(28) $$\frac{D-38.6}{36.6-955.3}=\frac{T-29}{-1}\;\Rightarrow \;\frac{D-38.6}{916.7}=T-29\;\Rightarrow \;D=916.7\cdot T-26623$$

**Figure 28:**
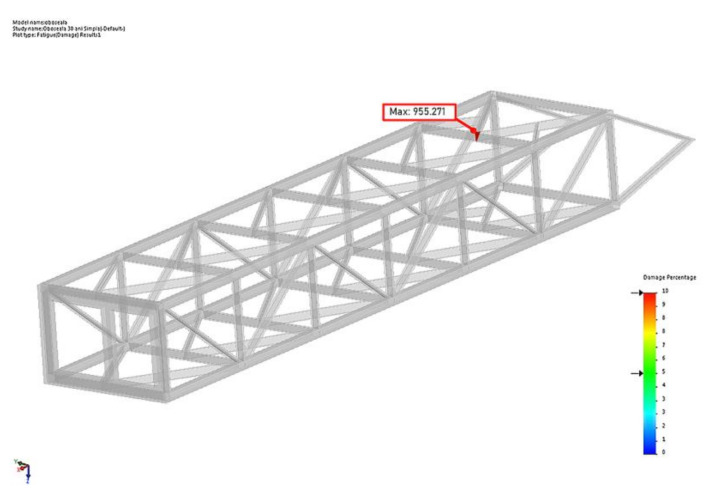
Damage min/max location for T = 30 years.

The equation is solved for the unknown T, thus it results that T_100_ = 29.15 years or 221.54 × 10^6^ events. Figure 28 presents the locations of damage min and max for 30 years.

## 5. Conclusions

The novelty of the approach is the combination of computer simulation for deriving the variable loads producing vibration, based on the technological forces during operation of BWEs, the time and frequency response, determination of the number of loading cycles, an experimentally determined SN curve, and the prediction of the remaining lifetime. Another new element of the study is that three positions of the boom were analyzed to find that the horizontal position of the boom is the most vulnerable to failure because of the vibrations given by excavation.

The results obtained are close to data on defects and failures occurring in excavators and similar equipment used in opencast mines, as recorded by the Oltenia Energy Complex and also in international literature, usually after 25 years of operation damage and failure probability is the highest.

Results are in line with research where the service life limit was predicted [41] using an expert assessment based on analogy to other machines and recorded data statistically processed, [42] where the fatigue prediction was based on experimental data and simulations, and [62] where similar periods are determined until failure.

It is important to note that the time until the damage is 100% obtained by simulation is under the estimated life time of a BWE as stated in [1], the DIN 22261-2 standard (paragraph 13) estimates the operational life to 50 years for the bucket wheel excavators, conditioned that periodic inspection is carried out including the measurement of strains in the nodes, and that the measured values are compared to those in the standard tables and meet the requirements.

By analyzing the graphic representations of frequency response of the BWE boom when subjected to the imposed excavations forces, the following can be concluded:

- The predominant response (displacement) is in direction *Y*, for the second modal frequency of 2.07 Hz, when the boom is in horizontal position;
- For direction *X* the maximum response corresponds to the first modal frequency and for direction *Z* the maximum response corresponds for the second modal frequency.

By analyzing the graphic representations of accelerations in the time response analysis during permanent excavation regime, it can be concluded that:

- All accelerations are variable in time and have an oscillating character;
- The greatest values of acceleration are obtained for direction *Y*;
- The acceleration for directions *X* and *Y* are symmetrical with respect to the time axis;
- The acceleration in direction *Z* is asymmetric with respect to the time axis.

Based on the displacements resulting after the simulation of the time response analysis during permanent excavation regime, it can be concluded that:

- All displacements are variable in time and have an oscillating character;
- The displacement has the highest amplitude in direction *Y*;
- Suppression of the excavation force determines a free vibration of the BWE boom that will be damped over time.

The results obtained on the studied type of BWE and the method itself are useful for the decision of maintaining in operation, replace or refurbish the equipment exceeding a certain number of operating hours, before the fault intensity increases dramatically. This type of fatigue analysis on a virtual model of a real structure, using the SOLIDWORKS Simulation package, can be adapted and applied to any kind of spatial structure subjected to vibrations that is built using CAD, using the same approach step-by-step, in order to obtain fatigue estimations. It can also be extended to other parts of industrial machines that can be modeled in a similar fashion.

## Figures and Tables

**Table 1:** Summary of the loads (static) exerted on the BWE boom, their magnitude and the SOLIDWORKS feature used to model them.

Static Load (Name)	SOLIDWORKS Feature	Magnitude (Unit)
Bucket wheel	Assembly	39.60 (tons)
Bucket wheel drive system	Mass (distributed)	29.00 (tons)
Hoisting ropes	Spring	35 × 10^6^ × 2 (N/m)
Discharge conveyor belt	Mass (remote)	25.00 (tons)

**Table 2:** Results of the simulation: Values of the maximum displacements and their corresponding frequency.

		Mode														
**Position**	**Result**	1	2	3	4	5	6	7	8	9	10	11	12	13	14	15
**(1)**	**f (Hz)**	1.74	1.91	4.63	5.32	6.66	8.15	9.92	12.51	12.80	13.64	16.49	16.76	16.87	17.08	17.15
	**max (mm)**	68.43	73.02	6.12	2.38	3.01	4.03	3.23	2.41	1.67	2.54	4.05	4.74	4.49	3.71	3.51
**(2)**	**f (Hz)**	1.77	2.00	4.68	5.33	6.66	8.14	9.94	12.53	12.79	13.66	16.49	16.76	16.87	17.08	17.16
	**max (mm)**	39.83	**92.6**	9.45	2.42	2.16	4.00	2.48	1.71	1.30	4.32	4.70	**5.48**	5.19	4.08	3.75
**(3)**	**f (Hz)**	1.76	1.91	4.64	5.32	6.65	8.14	9.93	12.52	12.78	13.65	16.49	16.76	16.87	17.08	17.16
	**max (mm)**	52.15	88.36	12.47	2.44	1.21	3.49	1.40	1.34	1.56	5.20	4.48	5.25	4.95	3.78	3.41

**Table 3:** Fatigue study results.

Damage (%)		Period Analyzed (years)	Events (×10^6^)
Min	Max		
2	13.20	10	76
2	19.60	15	114
3	26.20	20	152
3	27.40	21	159.6
3	28.80	22	167.2
3	30.30	23	174.8
3	31.40	24	182.4
4	32.90	25	190
4	34.10	26	197.6
4	35.50	27	205.2
4	36.70	28	212.8
4	38.60	29	220.4
5	955.30	30	228
